# Supplementary material for: Comparison of virtual reality development centers and 270-degree evaluations in the context of mid-level managers’ competencies
Source: PLoS One. 2026 Feb 6;21(2):e0339872. doi: 10.1371/journal.pone.0339872 (PMC12880633; doi:10.1371/journal.pone.0339872)
Supplement: S1 Data — (DOCX) [file pone.0339872.s001.docx]

|  | M managing people and task | | |  |  |  |  |  |  |
| --- | --- | --- | --- | --- | --- | --- | --- | --- | --- |
|  | G goal orientation | |  |  |  |  |  |  |  |
|  | D decision making process | | |  |  |  |  |  |  |
|  | CH change management | | |  |  |  |  |  |  |
|  | C Cooperation | |  |  |  |  |  |  |  |
|  |  |  |  |  |  |  |  |  |  |
|  | Evaluator person who evaluate managers- employee, supervisor | | | | | |  |  |  |
|  | AssessorVRDC1 First Assessor who evaluated managers in Virtual Reality Developemnt Center | | | | | | | | |
|  | AssessorVRDC2 Second Assessor who evaluated managers in Virtual Reality Developemnt Center | | | | | | | | |
|  |  |  |  |  |  |  |  |  |  |
|  | eM evaluator of the competency managing people and tasks  eG evaluator of the competency Goal orientation  eCH evaluator of the competency Change Management  eD evaluator of the Decision making  eC evaluator of Cooperation | | | | | |  |  |  |
|  |  |  |  |  |  |  |  |  |  |
