# Supplementary material for: Comparison of virtual reality development centers and 270-degree evaluations in the context of mid-level managers’ competencies
Source: PLoS One. 2026 Feb 6;21(2):e0339872. doi: 10.1371/journal.pone.0339872 (PMC12880633; doi:10.1371/journal.pone.0339872)
Supplement: S1 File — (DOCX) [file pone.0339872.s002.docx]

**SELF ASSESSMENT**

**Name and Surname: …………………………………**

**Evaluate how similar you are to a person who demonstrates the following behaviors in professional situations. Mark your response with an "X" on the five-point scale, where:**

1. **Completely dissimilar**
2. **Rather dissimilar**
3. **Neither similar nor dissimilar**
4. **Rather similar**
5. **Definitely similar**

**If you do not use such actions, select the "No data" column.**

**ASSESSMENT OF OTHERS**

**Name of the Assessed Individual: …………………………………**

**Evaluate the extent to which the assessed individual exhibits the following behaviors in professional situations. Indicate your response by marking "x" on the five-point scale, where:**

1. **Not at all**
2. **Rather not**
3. **Sometimes**
4. **Rather yes**
5. **Definitely yes**

**If you lack knowledge about the assessed individual’s actions in a specific area, or if the area does not apply to them (e.g., they do not perform such tasks), select the "No data" column.**

**QUESTIONNAIRE**

| **#** | **Behavior** | **1** | **2** | **3** | **4** | **5** | **No data** |
| --- | --- | --- | --- | --- | --- | --- | --- |
| **1** | **Plans work in such a way that others agree with the arrangements.** |  |  |  |  |  |  |
| **2** | **Takes the initiative in directing work.** |  |  |  |  |  |  |
| **3** | **Identifies opportunities and threats in the project being implemented.** |  |  |  |  |  |  |
| **4** | **Shows consistent energy in directing group work.** |  |  |  |  |  |  |
| **5** | **Effectively assigns roles, resources, and/or tasks.** |  |  |  |  |  |  |
| **6** | **Monitors the progress of work, ensuring tasks are completed on time.** |  |  |  |  |  |  |
| **7** | **Considers costs/losses/profits in proposed solutions.** |  |  |  |  |  |  |
| **8** | **Ensures work proceeds according to the plan unless unforeseen circumstances arise.** |  |  |  |  |  |  |
| **9** | **Rarely plans work. The team often does not know what to do.** |  |  |  |  |  |  |
| **10** | **Rarely takes the initiative.** |  |  |  |  |  |  |
| **11** | **Occasionally identifies opportunities and threats in the project being implemented.** |  |  |  |  |  |  |
| **12** | **Sometimes shows energy and initiative in directing group work.** |  |  |  |  |  |  |
| **13** | **Rarely assigns roles, resources, and/or tasks.** |  |  |  |  |  |  |
| **14** | **Monitors work progress spontaneously. Does not ensure tasks are completed on time.** |  |  |  |  |  |  |
| **15** | **Does not consider costs/losses/profits in proposed solutions.** |  |  |  |  |  |  |
| **16** | **Rarely monitors work progress.** |  |  |  |  |  |  |
| **17** | **Assigns tasks in a clearly defined order/timeframe. People know what follows next.** |  |  |  |  |  |  |
| **18** | **Shows persistence and determination in carrying out tasks.** |  |  |  |  |  |  |
| **19** | **Does not give up on tasks despite heavy criticism. Can persuade others to work towards goals.** |  |  |  |  |  |  |
| **20** | **Focuses on finding solutions in difficult situations.** |  |  |  |  |  |  |
| **21** | **When difficulties arise, focuses on the outcome, altering actions to achieve it fully.** |  |  |  |  |  |  |
| **22** | **Tries to maximize working time, usually achieving high-quality results.** |  |  |  |  |  |  |
| **23** | **Achieves set goals under significant time pressure.** |  |  |  |  |  |  |
| **24** | **Rarely assigns tasks in a clearly defined order/timeframe. People do not know what follows next.** |  |  |  |  |  |  |
| **25** | **Quickly abandons tasks when difficulties arise.** |  |  |  |  |  |  |
| **26** | **Gives up on tasks under criticism.** |  |  |  |  |  |  |
| **27** | **Focuses on finding those at fault in difficult situations.** |  |  |  |  |  |  |
| **28** | **Gives up on tasks when difficulties arise. Does not alter actions to try completing tasks.** |  |  |  |  |  |  |
| **29** | **Wastes time on unnecessary activities. Does not achieve high-quality results.** |  |  |  |  |  |  |
| **30** | **Rarely achieves set goals under significant time pressure.** |  |  |  |  |  |  |
| **31** | **Initiates changes when something is not working. Alters behaviors based on new information.** |  |  |  |  |  |  |
| **32** | **Uses persuasive/benefits-based language when communicating changes.** |  |  |  |  |  |  |
| **33** | **Is positively oriented towards new challenges/tasks.** |  |  |  |  |  |  |
| **34** | **Continues work when tasks suddenly change.** |  |  |  |  |  |  |
| **35** | **Does not try new approaches when something is not working. Does not alter behavior based on feedback.** |  |  |  |  |  |  |
| **36** | **Does not show benefits when communicating changes.** |  |  |  |  |  |  |
| **37** | **Is negatively oriented towards new challenges/tasks.** |  |  |  |  |  |  |
| **38** | **Stops actions when tasks suddenly change.** |  |  |  |  |  |  |
| **39** | **Strives to define the right problem. Accurately determines priorities.** |  |  |  |  |  |  |
| **40** | **Considers various solutions/options, effectively weighing pros and cons.** |  |  |  |  |  |  |
| **41** | **Does not agree with imposed problem frameworks. Shows multiple perspectives in analysis.** |  |  |  |  |  |  |
| **43** | **Rarely strives to define the right problem. Does not prioritize what is most important in the issue.** |  |  |  |  |  |  |
| **44** | **Does not consider various options. Focuses on the first solution they come up with.** |  |  |  |  |  |  |
| **45** | **Accepts the imposed problem framework. Does not show multiple perspectives in analysis.** |  |  |  |  |  |  |
| **46** | **Does not test how implemented solutions work. Does not draw conclusions from these trials.** |  |  |  |  |  |  |
| **47** | **Ensures smooth information flow.** |  |  |  |  |  |  |
| **48** | **Strives to alleviate conflicts, disputes, and emotions of others.** |  |  |  |  |  |  |
| **49** | **Introduces humor to diffuse tension and foster good relationships.** |  |  |  |  |  |  |
| **50** | **Encourages others to cooperate through their actions.** |  |  |  |  |  |  |
| **51** | **Willingly supports others in their actions.** |  |  |  |  |  |  |
| **52** | **Responds to proposed actions and ideas suggested by others.** |  |  |  |  |  |  |
| **53** | **Consults possible solutions with others.** |  |  |  |  |  |  |
| **54** | **Encourages team members to share their ideas.** |  |  |  |  |  |  |
| **55** | **Takes the initiative to help others. Is willing and open to cooperation.** |  |  |  |  |  |  |
| **56** | **Does not ensure smooth information flow.** |  |  |  |  |  |  |
| **57** | **Escalates or creates conflicts within the group.** |  |  |  |  |  |  |
| **58** | **Does not use humor to diffuse tension and foster good relationships.** |  |  |  |  |  |  |
| **59** | **Discourages others from cooperating through their actions.** |  |  |  |  |  |  |
| **60** | **Rarely supports others in their actions.** |  |  |  |  |  |  |
| **61** | **Ignores proposed actions or ideas from others.** |  |  |  |  |  |  |
| **62** | **Implements various solutions on their own without consulting others.** |  |  |  |  |  |  |
| **63** | **Does not encourage sharing ideas.** |  |  |  |  |  |  |
| **64** | **Does not take the initiative to help others. Is unwilling and not open to cooperation.** |  |  |  |  |  |  |

**ASSESSMENT OF OTHERS**

**Name of the Assessed Individual: …………………………………**

**Evaluate the extent to which the assessed individual exhibits the following behaviors in professional situations. Indicate your response by marking "x" on the five-point scale, where:**

1. **Not at all**
2. **Rather not**
3. **Sometimes**
4. **Rather yes**
5. **Definitely yes**

**If you lack knowledge about the assessed individual’s actions in a specific area, or if the area does not apply to them (e.g., they do not perform such tasks), select the "No data" column.**

**Supporting Information:**

**270-degree assessment questionnaire.
*Questionnaire used for the 270-degree assessment of managerial competencies applied in the study.***
