# Supplementary material for: Comparison of virtual reality development centers and 270-degree evaluations in the context of mid-level managers’ competencies
Source: PLoS One. 2026 Feb 6;21(2):e0339872. doi: 10.1371/journal.pone.0339872 (PMC12880633; doi:10.1371/journal.pone.0339872)
Supplement: Attachment — Submitted filename: Review of manuscript.docx [file pone.0339872.s003.docx]

**Review of manuscript titled:**

**Comparison of Virtual Reality Development Centers and 270-Degree Evaluations in the Context of Mid-level Managers’ Competencies**

**Authors of Article: Anna Katarzyna Baczynska; Zhenyao Cai; Lukasz Szajda; Konrad Urbanski**

1. **Summary of Research**

The authors correlated the ratings obtained from 270-degree assessments with the ratings obtained during a Virtual Reality Development Centre (VRDC) with each other. The ratings were for 64 individuals recruited from one organisation during a one-month recruitment period. The individuals attended 16 VRDCs. The five competencies assessed during the VRDC and the 270-degree assessments were *Managing People and Tasks*; *Goal Orientation*; *Decision-Making*; *Change Management*; and *Cooperation and Social Skills*.

The 270-degree assessment consisted of a *self-assessment*; and assessments from one manager and two co-workers based on 64 items in the assessment questionnaire using a 5-point rating scale. The ratings from the manager and the two co-workers were subjected to an inter-rater agreement test and only 52 individuals’ ratings correlated at an acceptable level and were used during the further analysis. The ratings of the manager and two co-workers were combined into “others assessment” and used during further analysis.

The scenario of the VRDC was “*Breakdown in the Powerplant*” and consisted of five stages: *Accessing the Control Room*; *Managing Critical Technical Parameters*; *Cooling System Management*; *Power Output Decisions*; *Crisis Resolution*. The two VRDC assessors’ rating per competency, per individual were also subjected to an inter-rater agreement test and combined for further analysis into “VRDC assessment”.

Both the “other assessment” and the “VRDC assessment” ratings were subjected to an analysis of variance (ANOVA) to examine between-group variations and compute the intraclass correlation coefficient (ICC). The analyses of variance, according to the authors, indicated that the results for both sets of ratings were within at least an acceptable range and that data aggregation was justified.

The relationship between this study’s variables (*self-assessment*; *others assessment* and *VRDC assessments*) were examined using bivariate correlations. The results strongly support hypothesis 3 that VRDC assessment ratings reveal insights into competencies not captured by 270-degree assessments. Results indicated partial support for hypothesis 1 which posited that there is a significant correlation between VRDC assessments and assessment ratings from 270-degree assessments. The results of the correlations indicated limited support for hypothesis 2 which proposed that VRDC assessment ratings would positively correlate with both self-assessment and other’s assessments. The authors stated that the results show the potential benefit of complementing 270-degree assessment results with VRDC results. They mentioned the small sample size, the homogeneity of participants and that the participants were all middle-level managers as limitations of the research constraining generalizability of the findings.

Although this reviewer is of the opinion that there are specific aspects in this manuscript that the authors should pay attention to, I appreciate that the authors have embarked on the process of researching the possible value that virtual reality simulations may bring to assessing competencies.

2. **Discussion of specific areas of improvement**

I recommend that the authors pay attention to the following:

2.1 *Introduction*

Using 360-degree assessment results as an additional source of information when implementing assessment centres (AC) for developmental purposes has long been a practice. The 360-degree assessment results indicate how the individual is experienced on-the-job, while the AC provides “a snapshot in-time” of the more objective measurement of the focal construct. Both these perspectives are incorporated when crafting a development plan for the individual.

This study brings into focus virtual reality simulations, a change to the delivery method of the assessment centre, while keeping constant the use of 360-degree assessments. The following are recommended:

- Expand the introduction of the manuscript to include a discussion on assessment centres and the advantage that virtual reality brings to assessment centres.
- Clearly describe what a virtual reality development centre is, as well as the potential advantages and disadvantages of designing, developing and implementing a virtual reality centre (i.e. advantages: potential realism; being able to potentially assess aspects that cannot easily be assessed by a digital / in-person assessment centre; disadvantages: Cost to design, develop, maintain; diversity and level of expertise required; etc.). In other words, clearly indicate the benefits of using virtual reality in ACs over digital / in-person ACs (make a business case).
- Link this study with research about 360-degree assessments and assessment centres (digital and / or in-person).
  2.2 *Materials and Methods*

A general critique on published articles in the field of ACs is that the articles do not report on all the variable aspects in ACs that impact the possible reliability an eventual validity of the AC (see Caldwell, C., Thornton III, G.C., & Gruys, M.L., 2003; Dewberry. C, & Jackson, D.J.R., 2016; Woehr, D.J., & Arthur, W. Jr., 2003). This creates a challenge for future AC research, especially meta-analysis on ACs.

Please describe the following aspects of the VRDC:

- The content and duration of the assessor training.
- The approach to behaviour observation, noting, classifying and evaluating: Checklist, or Behaviour Anchored Rating Scale (BAR), or Behaviour Observation Scale (BOS).
- Approach to integrating ratings per focal construct: Within Exercise Dimension Ratings (WEDR), or Behaviour Reporting Method.
- Clarify the rating scale that was used. It is stated that the scale used consisted of Level A; level B and level C. The scale was divided into smaller intervals, namely A=0; B=1; B=2; B+=3; C=4; and C=5 (p10). How was the scale practically used by the VRDC assessors; as well as the 270-degree assessors?
- Were all five competencies assessed during all five stages of the VRDC scenario?

Please also describe the training of the 270-degree assessors related to the use of the rating scale; the understanding of the competencies; and the period to consider.

The VRDC and the 270-degree assessment instrument evaluated participants behaviour on five competencies. Where do these competencies come from? Is it based on a job analysis, or a competency framework of the client organisation?

2.3 *Results*

The results of the interrater agreement for both the VRDC and the 270-degree assessments were mentioned in the manuscript, as well as the ICC values. Perhaps tables indicating these results can be included to support the findings by the authors.

Five tables indicating the results from a bivariate analysis per competency, correlating the VRDC rating; the Self-rating and the Other-rating were included. Each time comments about the interpretation of the results were given. Please add additional comments to the interpretation of each of the five tables. As example, on Table 1 (p11) “Regarding the evaluation of M, employee self-assessments were significantly and negatively correlated with VRDC assessments (r = -0.29, p < 0.05), and others assessments were significantly and positively correlated with VRDC assessments (r = -0.40, p < 0.01).” Add what this means for the individual, as example: the individuals themselves under evaluate their capability on this competency when compared to the VRDC rating, while Others’ rating tends to be more aligned to the VRDC rating. (Note that in the text on p11 the Other-rating r=-0.40, while in Table 1 (p12) it is indicated as 0.40.)

Perhaps also specify which bivariate method was used.

It could perhaps also be useful to add a chart (like a bar-chart) to visually indicate the correlation of the VRDC ratings with the Self and Other ratings per competency. As example:

(example chart compiled using AI software)

**Please note that I am not an expert in statistical analysis and defer to experts**

2.4 *Discussion*

By adding more research article citations to the discussion in the manuscript’s *Introduction* and expanding that discussion to include research on ACs and 360-degree assessments, would facilitate linking the results from this study to existing research findings.

Please add a citation for the following statement: “While traditional methods like 270-degree evaluations excel in capturing long-term and relational competencies…..”

On p15 the following is stated: “Longitudinal studies could also examine whether VRDC assessments translate into sustained improvements in employee performance over time.” What does the existing research state about translating assessment results into sustained improvements (Bailey & Austin, 2006; Camp, Blanchard & Huszczo, 1986; Kudisch, Lundquist, & Smith, 2002 in Woo et al., 2008; Thornton, Rupp, & Hoffman 2015; Woo, S.E., Sims, C.S., Rupp, D.E., & Gibbons, A.M. 2008)?

2.5 *Conclusions*

Although I concur with most of the content in this section of the manuscript, I will recommend that “VRDC assessments *correlate significantly* with 270-degree evaluations in areas such as managing people and tasks, goal orientation, and change management, validating its reliability for these competencies” be adapted to reflect that self-ratings are mostly misaligned to the VRDC ratings and the other ratings are moderately related to the VRDC ratings, except on change management where it is significantly related (however, I am not an expert on statistical analysis and defer to the experts opinion).

2.6 *References*
- Expand the reference list by citing more articles to support this study. As example, instead of only citing one article to support a finding in the manuscript, cite more published peer review articles as support. Currently only 16 references are provided in the reference list.
  2.7 *Overall*
- Please ensure that all statements made in the manuscript are supported by applicable citations. As example: “Competency assessment is a key component of human resource development strategies, directly impacting individual career growth and organizational success.” – citation required. “Unlike traditional evaluations, VRDC minimizes subjectivity and enhances competency assessment through controlled, high stakes simulations.”- citation required.
- Be careful of using adjectives that may only reflect the opinion of the authors. As example: “*innovative* tool” (p2); “*revolutionize* organizational assessment and development practices” (p15).
  2.8 Other Comments

To be accepted for publication in *PLOS One*, research articles must satisfy the following criteria:

1. The study presents the results of original research. *This is original research*
2. Results reported have not been published elsewhere. *I could not find that this study was published elsewhere*
3. Experiments, statistics, and other analyses are performed to a high technical standard and are described in sufficient detail. *This is an area needing attention – see above*
4. Conclusions are presented in an appropriate fashion and are supported by the data. *This needs attention – see my comment above.*
5. The article is presented in an intelligible fashion and is written in standard English. *Yes, it is written in standard English.*
6. The research meets all applicable standards for the ethics of experimentation and research integrity. *Yes, according to my knowledge, it does*.
7. The article adheres to appropriate reporting guidelines and community standards for data availability. *The authors mentioned that the data is available, I did not access the data, and I made recommendations about reporting the data.*
