# Supplementary material for: Comparison of virtual reality development centers and 270-degree evaluations in the context of mid-level managers’ competencies
Source: PLoS One. 2026 Feb 6;21(2):e0339872. doi: 10.1371/journal.pone.0339872 (PMC12880633; doi:10.1371/journal.pone.0339872)
Supplement: Attachment — Submitted filename: Review Report, Plos One.docx [file pone.0339872.s004.docx]

**Comments to the Author**

Thank you for reviewing your manuscript, which is aimed at Comparison of Virtual Reality Development Centers and 270-Degree Evaluations in the Context of Mid-Level Managers' Competencies. While I believe the fundamental premises of the paper are worthy research endeavors, a number of critical issues loom large. In particular, the most significant concerns related to its contribution and its value-added beyond what we already know and the methodology employed.

However, I encountered some discrepancies that can enhance the chances of publication at a later stage.

**Abstract:**

- First of all, try to improve the abstract and make it more compelling by emphasizing research design and the theoretical framework. The current version lacks to highlight the study's potential gap and actual contribution. So I suggest the authors revise the abstract to consider its theoretical significance, not just its empirical contribution.

**Introduction:**

- The start of the introductions is highly weak. For instance, authors discuss virtual reality but fail to support their arguments with reference to previous literature. Although the term is not new.
- The authors must outline in more detail the study's contributions in the last paragraph of the introduction. Currently, the study highlights the author's discussion of narratives that emphasize the actual contribution. Hence, authors need to be more specific in showcasing their contribution to the various bodies of literature. I highly recommend that authors justify their contributions in light of recently published studies.
- I suggest reworking the introduction, as it is currently too far from the main theme, leading to confusion about your study and its contribution to our knowledge.

**Method:**

- The authors utilized the headings of "Participants and Procedure,". The authors should first provide a detailed discussion of the sample and procedure before addressing the response rate or total number of respondents, rather than mentioning these figures upfront. Additionally, they need to clarify how respondent confidentiality was ensured. This section is not properly written.
- Also, support your measurement scales in light of previous investigations in the public sector.

**Discussion:**

- While the paper provides a detailed elaboration of results and justifies them with other elements, I suggest adopting a more critical approach in compiling the findings and discussion, considering the theoretical framework and recent literature. Moreover, the discussions should be aligned with each research question to better portray the results to readers.
- The authors should add separate headings for "Implications" to provide clearer guidance for the reader. The "Conclusion" should also be a distinct section that includes limitations and suggestions for future research.
